# Heal Thyself to Heal and Cure: Voices of Women in Cardiology

**DOI:** 10.1016/j.jaccas.2021.07.020

**Published:** 2021-09-15

**Authors:** Kamala P. Tamirisa, Laxmi S. Mehta, Smadar Kort, Marci Farquhar-Snow, Ami B. Bhatt, Jane A. Linderbaum, Hena Patel, Christina Cardy, Gina P. Lundberg, Sherry Ann Brown

**Affiliations:** a Texas Cardiac Arrhythmia Institute, Dallas, Texas, USA; b Division of Cardiology, Ohio State University Wexner Medical Center, Columbus, Ohio, USA; c Division of Cardiology, Stony Brook Heart Institute, Stony Brook, New York, USA; d Division of Cardiology, Mayo Clinic, Scottsdale, Arizona, USA; e Division of Cardiology, Massachusetts General Hospital, Boston, Massachusetts, USA; f Division of Cardiology, Mayo Clinic, Rochester, Minnesota, USA; g Division of Cardiology, University of Chicago School of Medicine, Chicago, Illinois, USA; h Division of Cardiovascular Sciences, University of South Florida, Tampa, Florida, USA; i Division of Cardiology, Emory University School of Medicine, Atlanta, Georgia, USA; j Division of Cardiology, Medical College of Wisconsin, Milwaukee, Wisconsin, USA

**Keywords:** burnout, COVID-19, self-care, well-being, ACC, American College of Cardiology, COVID-19, coronavirus disease-2019, PPE, personal protective equipment, WHO, World-Health Organization

*After all, we are humans first and cardiovascular clinicians second.*

The coronavirus disease-2019 (COVID-19) pandemic has highlighted the need to focus on psychological and physical well-being as cardiovascular clinicians who care for patients with inevitably higher rates of morbidity and mortality. The pandemic has offered us a unique opportunity to redress the concept of personal well-being. It is well known that clinicians’ well-being is linked to patients’ outcomes and cost containment. While organizations need to address systemic issues that impact well-being, creative skills can enhance the clinician’s ability to confront stress and burnout along their career by developing behaviors that adjust to new or challenging situations. Individuals should partake in hobbies and mindfulness activities to improve their personal resiliency. Arts have a significant impact on mental and physical health according to the World Health Organization Health Evidence Network Synthesis report (1). In this paper, we share various strategies (Figure 1) we have used as women in cardiology as approaches towards self-care and well-being.

**Figure 1:**
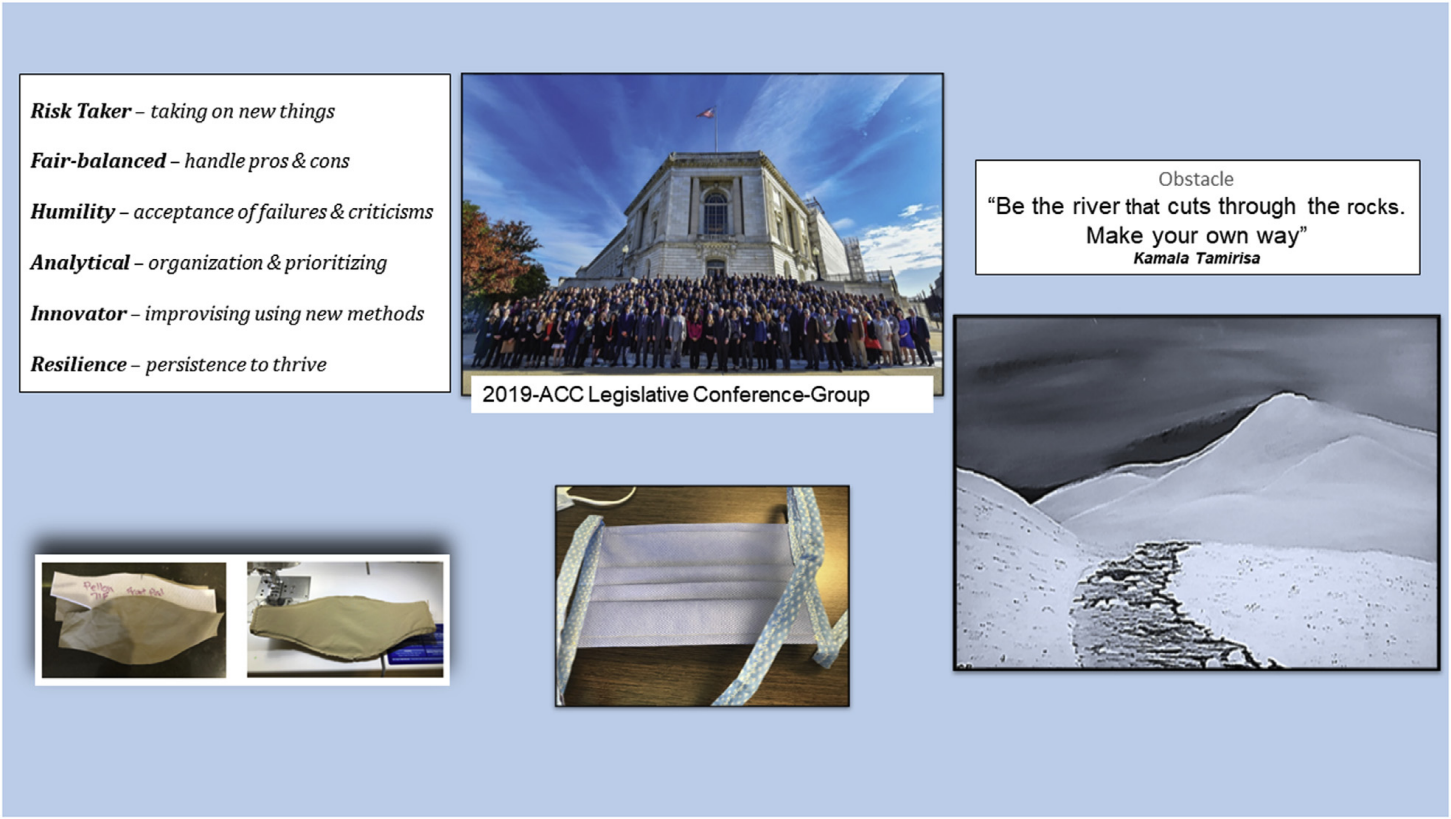
Creative Paths to Well-Being 2019 Legislative Conference (photo courtesy Bill Petros Photography).

## Paths to Well-being

### Mindful canvas

“Medicine and art have a common goal: Art like medicine, is not an arrival: it’s a search. This is why, perhaps we call medicine itself an art” (2).

The practice of cardiology and painting on canvas can be complementary, one surrounds human suffering, and the other offers a refuge from the same. The roller coaster of a daily routine of sickness and unpredictable outcomes can take us from the highs of purpose to the depths of helplessness, leaving a void that begs for restoration and rejuvenation that makes us humans. Painting can help renew the human spirit within and close the loop of creation and healing. The necessary steps of planning and meticulous mixing of colors to deliver the finished "masterpiece" remind us to slow down to achieve more. Apart from being an avenue for recharge that has been much needed during COVID-19, painting can also be self-empowering, and a tool for networking—perhaps a journey of inspiration to others.

### Power of the pen

In medicine, the most trying days and the patients’ stories that live on in our hearts become an integral part of us. Allowing time for self-contemplation allows us to build on our viewpoints and have greater knowledge of what is genuinely inside of ourselves. Cultivating a form of creative expression through journaling and poetry enables us to not only communicate what is within, but also to solidify what is floating around in our minds, hearts, and souls. This can benefit us, as well as those around us. Those who read our work might find themselves reflected in what we write. Thus, while we pour so much of ourselves out for our patients—when we write, we get to pour so much of ourselves out for our own selves, which is invigorating for us and others we care for. Here is an excerpt written by a cardiologist during COVID-19:

“And the people went to work And wore masks and paper gowns And cared for the feverish and weak… And tried to give comfort” (3)

### Mend with compassion

Sewing and crafting skills can promote self-relaxation and reduce stress. Sewing can teach us to develop self-care and resiliency, especially when encountering challenging situations. We can nurture humility, analytical thinking, innovation, and improvisation. For example, during the COVID-19 pandemic, volunteering to sew masks for the public stimulated creativity while addressing the immediate health care goals to educate and prevent viral dissemination. These skills also supported our ability to empathize with others.

### Culinary art

Meditation can help to quiet the mind and focus on being present. Cooking is an unconventional form of guided meditation that produces a tangible and potentially delightful result. With the cookbook serving as a guide, the chef can detach from responsibilities of work while maintaining a sense of control as they choose a recipe, prepare the food, and follow the steps to achieve the finished product. A person’s external environment can mirror their inner being, and disorganization in the kitchen can exacerbate feelings of stress. After enjoying the meal, with family or friends, the process of cleaning the kitchen may leave the chef with feelings of satisfaction, offering a blank slate of new possibilities for future fares. Cooking is a therapeutic exercise that stimulates creativity while nourishing the body and soul.

### Social connectedness

Social connectedness is a fundamental need. Adverse social markers such as loneliness reduce longevity more than obesity, alcohol, use or smoking (4). After COVID-19, dissociation from friends, family, and coworkers has led to dwindled relationships for many. Depleting social connectedness adds to physical and mental exhaustion and reduced self-worth. Conversely, social connectedness creates a supportive environment that protects from burnout while building stronger self and community. A few mechanisms to encourage social connectedness include: 1) the creation of health care teams with decreased hierarchical structure to offer a chance for shared purpose; 2) promoting self-empathy with supportive and forgiving camaraderie to create well-being culture; and 3) avenues to engage in new skill development leading to positive social culture and better retention. Social connectedness is the essence of individual happiness and higher organizational performance for better clinician and patient outcomes.

### Fitness attitude

Despite the well-established health benefits of exercise, maintaining a regular exercise program can be difficult at the best of times, let alone in the midst of the conditions surrounding the pandemic. As the COVID-19 stressors inundated health care systems, physical activity has become an important strategy for interrupting the stress cycle. This challenging time has been an opportunity to explore new ways to stay active. Online classes, fitness apps, and virtual workout groups have grown in popularity. Indeed, delineating time for exercise can help regain a sense of control in daily life by pursuing gardening, dancing, walks, and so on. The built-in social connection of many fitness platforms can increase the likelihood of successful commitment. Physical fitness is an essential component of well-being.

### Voice of advocacy

Women excel in negotiating on behalf of others. Maternal instincts and striving for fairness make them strong negotiators and advocates. Using these skills during the COVID-19 pandemic and advocating on behalf of staff and colleagues has served as a way to relieve stress. Protecting the people around us by creating clinical protocols that minimized exposure, and negotiating with the administration for proper personal protective equipment paved the way for a greater sense of self-worth. Being active in the American College of Cardiology’s advocacy efforts at both the local and the national level have been personally and professionally gratifying. Legislation regarding telehealth and mental health support for health care workers are essential and are key American College of Cardiology advocacy initiatives that have been supported and advanced by women in cardiology. Advocating on behalf of our profession and our patients helps us to support others even beyond COVID-19.

### Contributing to medical organizations

Volunteerism can be an opportunity to contribute one’s time and talents without expectation for benefit or compensation. Medical mission trips and community service are examples of how health care professionals can contribute to the well-being of oneself and teams. Often this requires personal knowledge, skills, and attributes applied in clinical scenarios much different from typical professional practices. The ability to contribute to community well-being provides a sense of gratitude, insight, and opportunity. Medical organizations are challenged to provide cost-effective care, particularly in underserved areas. Medical and community organizations depend on volunteerism of medical professionals for successful care delivery. Patients, communities, and health care workers join hands with shared vision and contribute to improve the well-being and health of individuals and communities. These experiences often benefit the volunteers as well as those served.

## Conclusions

COVID-19 was an unprecedented challenge to cardiovascular clinicians, including female professionals. Exhausting long days spent caring for patients during the pandemic provided a completely new experience that no one had predicted, going beyond what all of us had trained for. While our lives outside of medicine came to a near complete stop, our work lives escalated in ways we never could have imagined. Finding ways to de-stress, rejuvenate, and become resilient were varied and personal. Some of us found comfort in old hobbies that we had not engaged in for years. Others found new ways to engage in personal free time and temporarily feel distracted from the stress of COVID-19. We must continue to cultivate downtime and unplug from the grinding routine and make time for creative escapes to heal ourselves if we wish to resist burnout and be fully present and available to our patients, loved ones, and ourselves.

## Funding Support and Author Disclosures

The authors report that there are no relationships relevant to the contents of this paper to disclose.
